# A. Case of Ovariotomy

**Published:** 1853-09

**Authors:** J. Taylor Bradford

**Affiliations:** Augusta, Kentucky


### WESTERN JOURNAL

OF

### MEDICINE AND SURGERY.

SEPTEMBER, 1853.

---

ARTICLE I.—*A Case of Ovariotomy.* By J. TAYLOR BRADFORD, M.D., of Augusta, Kentucky.

The subject of this operation was Miss Nancy Harrison, of Mayslick, an unmarried young lady twenty-one years of age. At the time the tumor appeared she was but little over nine years old. It was felt in the left groin, and then appeared to be about the size of a goose's egg. About three years after the tumor was perceived, the menstrual flux occurred, and continued to return at pretty regular intervals. The progress of the tumor was watched by Dr. Basil C. Duke, of Mayslick, the intelligent physician of the family, who says; "When I became satisfied that it was a case of ovarian tumor I insisted upon Miss H.'s visiting some of the most distinguished surgeons, and I visited Lexington in company with her. She was advised by all of those to whom she applied not to submit to the operation, as they looked upon it as hopeless."

When I was sent for to visit her she had just returned from Lexington. On examination, I found the walls of the abdomen so much distended by the enormous tumor that very little, if any, movement of the foreign body could be effected. Upon the anterior superior part of the tumor above the umbilicus could be felt a hard substance, evidently imbedded in the sac. It seemed to be about the size of the hand, and suggested the idea of bone. I thought in particular positions, and by certain manipulations, it was slightly moveable,gliding under the parietes of the abdomen.

Assisted by Dr. Dunlap, of Ripley, Ohio, and in presence of Dr Duke, who aided us by judicious suggestions, on the 14th of last June, I proceeded to remove the tumor. The patient was subjected to the influence of chloroform—not sufficiently to induce profound sleep, but to annul pain. The pulse did not vary ten in the minute from the commencement of the operation till six hours after it was completed. An incision of fifteen or eighteen inches was required for the extraction of the tumor. At first it was carried to the extent of about four inches down to the tumor, and then extended downwards to the pelvis, and upwards two inches above the umbilicus, making some twelve or fourteen inches. Finding that a strong adhesion to the omentum existed at the upper part of the tumor, I was obliged to carry the incision three or four inches higher. The bands connecting the tumor to the omentum proved to be large and very firm, and were inserted by several points into the bony substance which had been recognised before the operation. It required considerable force to break up the adhesion, which was done by the fingers and the handle of the scalpel. This procedure gave rise to very little hemor rhage. The sac was now punctured, and after drawing off about three gallons of fluid, we attempted to raise the tumor out of the abdomen, but finding it to be too heavy to handle, we determined to draw off what fluid still remained in it. This being done we were able to reach the base of the tumor, at which there were no serious adhesions. Lifting the tumor from the cavity, the pedicle was transfixed with a needle, armed with four strands of saddler's silk; the ligature was then divided at the eye of the needle, each segment of the pedicle was tied securely, and the tumor extirpated about two inches above the ligature.

The appearance presented by the contents of the abdomen, on the removal of the tumor, was striking and exceedingly interesting. The liver, the stomach, the spleen, and the intestines, were all in full view under the eye, and in a perfectly healthy condition. It is the first time that I had witnessed such a spectacle in the living human body. The viscera were not disturbed further than was requisite for sponging out the blood and little liquid which had insinuated themselves among them.

On examination of the tumor, the hard substance spoken of as felt through the walls of the abdomen proved to be bone. It was contained within the sac in front, and extended throughout its entire length. It consisted of scales of bone varying in size from that of the thumb nail down to that of a pin's head. The surface of the sac on the inner and front part was rugose and uneven, whilst the part lying next to the back was smooth, without any appearance of osseous degeneration. At the bottom of the tumor, lying in the pelvis, there were several small fleshy tumors of various sizes, from that of a cocoa nut to that of a hen's egg. On cutting into these tumors a little fluid escaped, and within each one there were found to be a series of still smaller sacs of various shapes. The most diminutive of these groups of cells were so small as scarcely to be visible to the unassisted eye. The principal sac, bony substance, fleshy part of the tumor, and liquid, all, weighed forty-one pounds and eight ounces.

The patient had a quick recovery, without a single unpleasant symptom. The operation required twenty-four minutes. In six weeks from the time it was performed the young lady, who had been twelve years burdened with the tumor, was riding about returning calls.

JULY 21ST, 1853.

